# ZnO-decorated SiC@C hybrids with strong electromagnetic absorption

**DOI:** 10.3762/bjnano.14.47

**Published:** 2023-05-04

**Authors:** Liqun Duan, Zhiqian Yang, Yilu Xia, Xiaoqing Dai, Jian’an Wu, Minqian Sun

**Affiliations:** 1 State Key Laboratory of Disaster Prevention & Mitigation of Explosion & Impact, Army Engineering University of PLA. Nanjing 210007, P.R. China

**Keywords:** carbon, dielectric, electromagnetic absorption, SiC nanowires, ZnO

## Abstract

A novel strategy is provided to improve the absorption of SiC nanomaterials through surface carbonization of SiC nanowires and hydrolysis. SiC@C-ZnO composites were synthesized with different dosages of ZnNO_3_·6H_2_O. Composition, microstructure, and electromagnetic properties of the composites were characterized and analyzed. Results from TEM and XRD show that crystalline ZnO particles adhere to the surface of amorphous carbon, and the ZnO content increases as a function of a dosage of ZnNO_3_·6H_2_O. The as-prepared SiC@C-ZnO hybrids exhibit effective electromagnetic absorption, which is related to a synergy effect of different dielectric loss processes. The minimum reflection loss reached −65.4 dB at 11 GHz at a sample thickness of 3.1 mm, while the effective absorption bandwidth (EAB) reached 7 GHz at a sample thickness of 2.56 mm. Furthermore, the EAB of the samples can also cover the whole X band and Ku band at small sample thicknesses (2.09–3.47 mm). The excellent properties of the materials suggest great prospect as electromagnetic absorbers.

## Introduction

With increasing functionality of electronic devices, the widening of the working frequency bands, and the diversification of working conditions, new electromagnetic (EM) absorbing materials are gradually designed and fabricated to obtain thinner, lighter, wider, and stronger materials than the traditional materials such as carbonyl iron and ferrite [1–4]. SiC has the advantages of low density, high-temperature stability, chemical corrosion resistance, and high strength. Hence, it has received a lot of attention [5–7]. Because of the high resistivity and the low dielectric parameters of traditionally prepared SiC, its EM absorption is poor [8]. According to existing literature reports, the electromagnetic parameters of SiC can be effectively adjusted by means of morphology design [9–11], doping [12–14], and surface modification [15–17], thereby improving their EM absorption properties. However, the design of SiC-based absorbers with relatively high reflection loss (RL_min_ < −60 dB) and, at the same time, wide effective absorption bandwidth (EAB ≥ 7 GHz) remains a great challenge. A good strategy is to form hierarchical heterostructures, characterized by diverse components, abundant heterogeneous interfaces, multiple reflective paths, and enrichment of structural defects [18–20]. Nevertheless, the EM absorption of most SiC-based absorbers with heterostructures is far from satisfactory [21–23].

In our previous work, SiC@C nanowires have been successfully obtained by surface carbonization of SiC nanowires [24]. Carbon materials are prone to bond with other dielectric or magnetic materials due to their high content of surface functional groups. Among dielectric materials, zinc oxide possesses the outstanding characteristics of low cost, non-toxicity, excellent thermodynamic stability and photostability, and unique semiconducting properties. Hence, it is widely used in the fields of photocatalysis, adsorption, and EM absorption [25]. Researchers have developed ZnO-based absorbing materials with different microstructures, such as core–shell structures [26], flower-like structures [27], rod-like structures [28], cage-like structures, and nanoparticles [29–30]. Wu et al. demonstrated that it is a good strategy to grow ZnO nanocrystals on the surface of SiC nanowires. The final hybrids (SiC@ZnO) showed enhanced performance, with a higher value of effective absorption bandwidth (EAB = 6.6 GHz) and lower RL_min_ (−42.11 dB) [16]. Growing ZnO nanocrystals on carbon materials (e.g., MWCNTs or graphene) may be easier because of the abundance of oxygen functional groups (e.g., carboxyl or hydroxy groups) on the carbon surface [26,31–32], in comparison to pure SiC. The introduction of carbon may help to further adjust the electromagnetic parameters and performance of SiC@ZnO nanocomposites, which has been rarely investigated before.

Herein, we describe a new strategy for preparing ternary hybrids (SiC@C-ZnO, SCZ) by growing ZnO particles on carbon surfaces derived from SiC nanowires. The influence of ZnO precursor (ZnNO_3_·6H_2_O) dosage on composition, microstructure and electromagnetic properties of the SCZ samples is discussed in detail.

## Experimental

### Preparation of SiC@C nanowires

The synthesis of SiC@C was described in our previous work [24]. The synthesis temperature has been fixed to 800 °C for 1 h for the carbonization of SiC_nw_.

### Fabrication of SiC@C-ZnO hybrids

Different amounts of ZnNO_3_·6H_2_O (0.5, 1, 2, 3, and 4 mmol), 50 mL H_2_O, and 8 mL PEG were mixed together. After that, 40 mg SiC@C nanowires was added to the above solution, followed by magnetic stirring for 20 min. At 60 °C, NH_3_·H_2_O was added dropwise until pH 9–10. After 2 h of continual stirring, the products were obtained after filtering, washed with distilled water, and dried at 50 °C under vacuum. The final samples were obtained by calcination at 600 °C for 4 h in a tube furnace (Ar, 99.999% purity). We labeled the samples as SCZ*x*, where *x* indicates *x* mmol dosage of ZnNO_3_·6H_2_O.

## Results and Discussion

**Figure 1:**
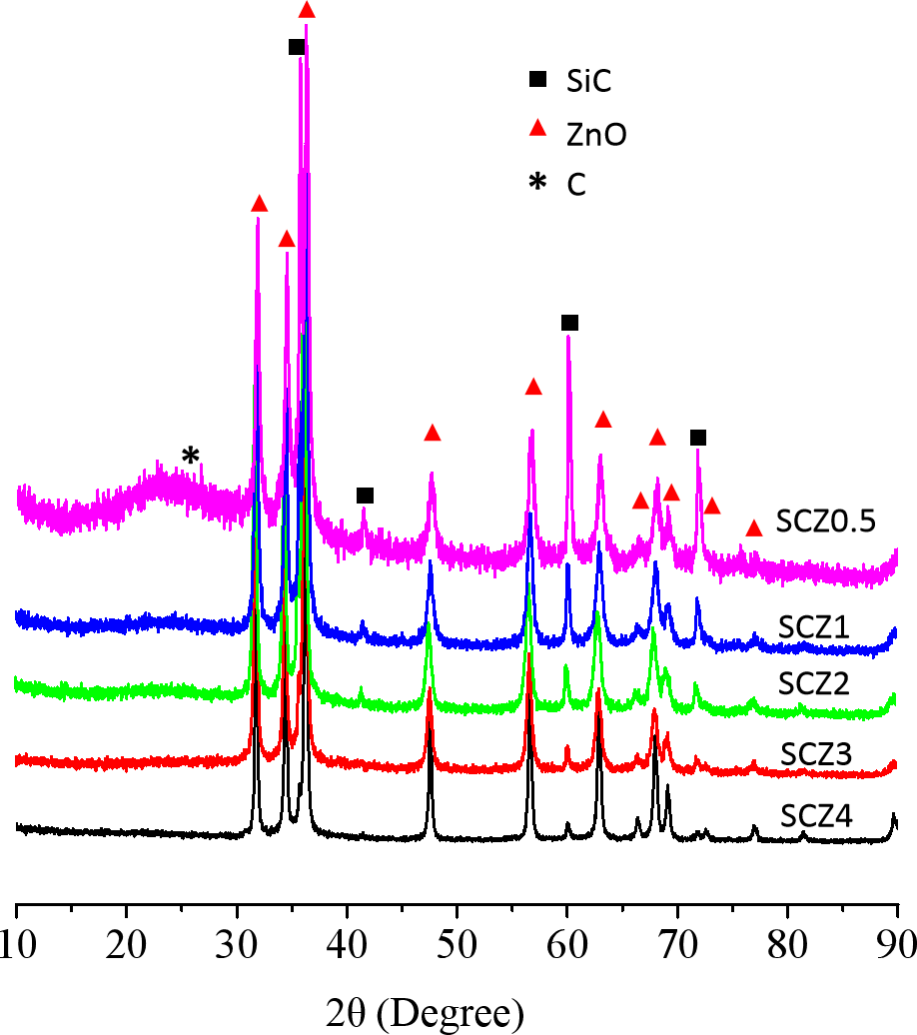
XRD diffractograms of all samples.

All SCZ samples exhibit several strong XRD reflections (31.81°, 34.41°, 36.21°, 47.51°, 56.61°, 62.81°, 66.41°, 68.01°, 69.11°, and 76.91°), corresponding to the (100), (002), (101), (102), (110), (103), (200), (112), (201), and (202) planes of ZnO (hexagonal structure, PDF#36-1451), respectively (Figure 1). Besides, some sharp diffraction peaks (35.65°, 41.40°, 60°, 71.78°, and 75.5°) can be detected, which point to the (111), (200), (220), (311) and (222) of β-SiC, indicating that the pristine SiC phase was maintained after surface carbonization and hydrolysis. The relative intensity of the SiC diffraction peaks gradually gets weaker with increasing added amounts of ZnNO_3_·6H_2_O. Also, a small peak at 26.5°, which corresponds to the (002) reflection of amorphous carbon, can be found in the SCZ samples, especially when the dosage of ZnNO_3_·6H_2_O is low. A carbon phase on the SiC surface was obtained through the removal of Si atoms from SiC_nw_ in the carbonization atmosphere containing a low concentration of chlorine gas [24].

**Figure 2:**
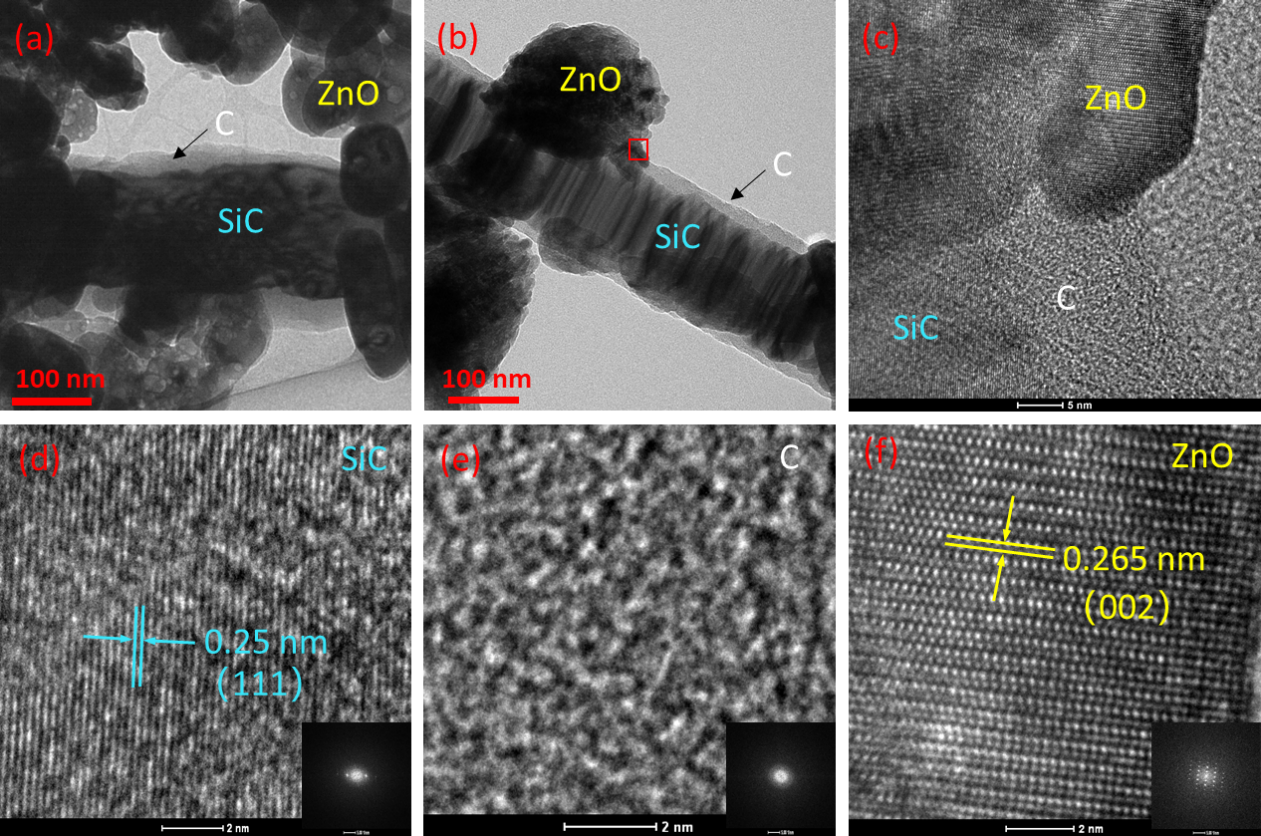
TEM images of all samples. (a) SCZ4; (b–f) SCZ3; (c–f) HRTEM images with corresponding FFT images derived from the red box area in image (b).

Figure 2 shows TEM and HRTEM images of the final SiC@C-ZnO samples. The SCZ samples are composed of SiC, carbon, and ZnO particles. Obviously, the hybrids are characterized by SiC cores and carbon shells, as well as ZnO particles growing randomly on the outside (Figure 2a,b). It can be observed that an increasing dosage of ZnNO_3_·6H_2_O will lead to an increase in the density of ZnO particles on the carbon structure (Supporting Information File 1, Figure S2), which agrees with the XRD results.

Figure 2c clearly shows the two kinds of interfaces, that is (1) the interface between a SiC core and a carbon shell and (2) the interface between the carbon phase and a ZnO particle. The (111) and (002) interplane spacings of, respectively, β-SiC and ZnO can be seen (Figure 2d,f), while the carbon is an amorphous state (Figure 2e). The carbon shell may have a positive effect on the nucleation of ZnO particles. This is because oxygen-containing functional groups (such as carboxyl and hydroxy groups) and structural defects are generated on the SiC@C surface during the in situ carbonization [24], which both provide locations for the deposition of Zn^2+^ via electrostatic interactions. Cao et al. [26] have reported the growth of ZnO particles on MWCNTs through a similar mechanism. However, in their case, the functional groups on the MWCNTs were obtained by ultrasonic treatment in concentrated nitric acid.

**Figure 3:**
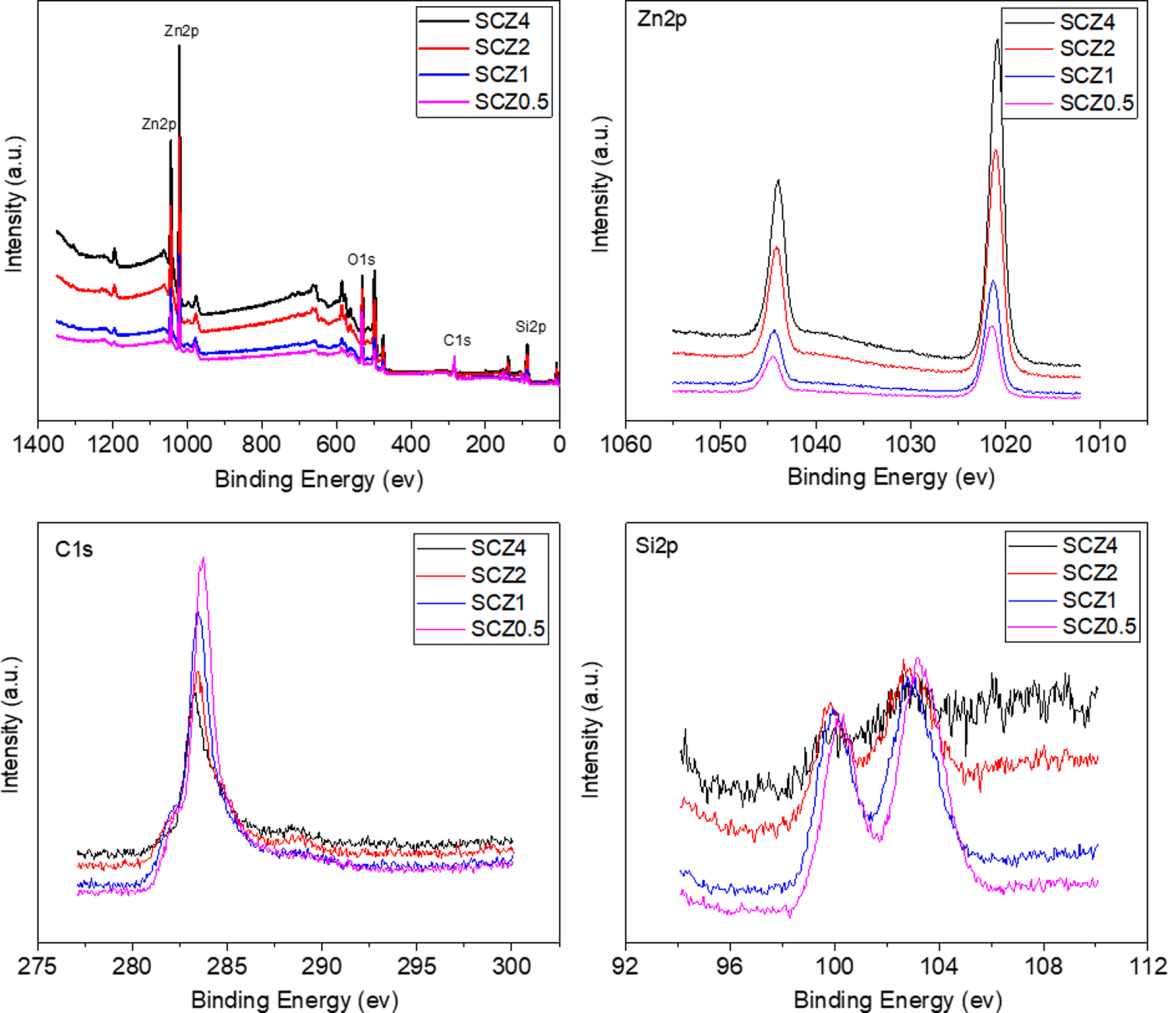
XPS survey spectra of SCZ samples.

Four elements (C, Si, O, and Zn) have been determined by XPS in all SCZ samples, as shown in Figure 3. The survey scans of the SCZ samples exhibit major peaks assigned to C 1s (ca. 284 eV), O 1s (ca. 532 eV), Si 2p (ca. 100 eV), Zn 2p (ca. 1022 eV and ca. 1045 eV). The two Zn 2p peaks correspond to Zn 2p^3/2^ and Zn 2p^1/2^, respectively. This proves that Zn exists in the form of Zn^2+^ [33]. Besides, it is obvious that the relative intensity of the two Zn 2p peaks gradually increases as a function of the ZnNO_3_·6H_2_O dosage, suggesting an increasing fraction of ZnO in the samples. This is consistent with the TEM results. In contrast, the relative intensities of the Si 2p and C 1s peaks gradually decrease at the same time. The peaks at 100 and 103.5 eV are related to Si 2p_1/2_ and Si 2p_3/2_, respectively. Table 1 provides the elemental compositions (atom %) on the SCZ sample surfaces as determined by XPS. The fractions of O and Zn increase with increasing dosage of ZnNO_3_·6H_2_O. Simultaneously, the fractions of C and Si decrease. It can also be found that the atomic ratio of C is larger than that of Si, indicating that C does exist in the form of free carbon in addition to SiC.

**Table 1:** Elemental analysis of SCZ samples via XPS.

Sample	Si (atom %)	O (atom %)	Zn (atom %)	C (atom %)

SCZ4	1.90	39.97	34.23	23.90
SCZ2	9.03	40.53	23.06	26.60
SCZ1	14.83	37.50	11.99	34.65
SCZ0.5	16.91	36.78	7.95	38.36

**Figure 4:**
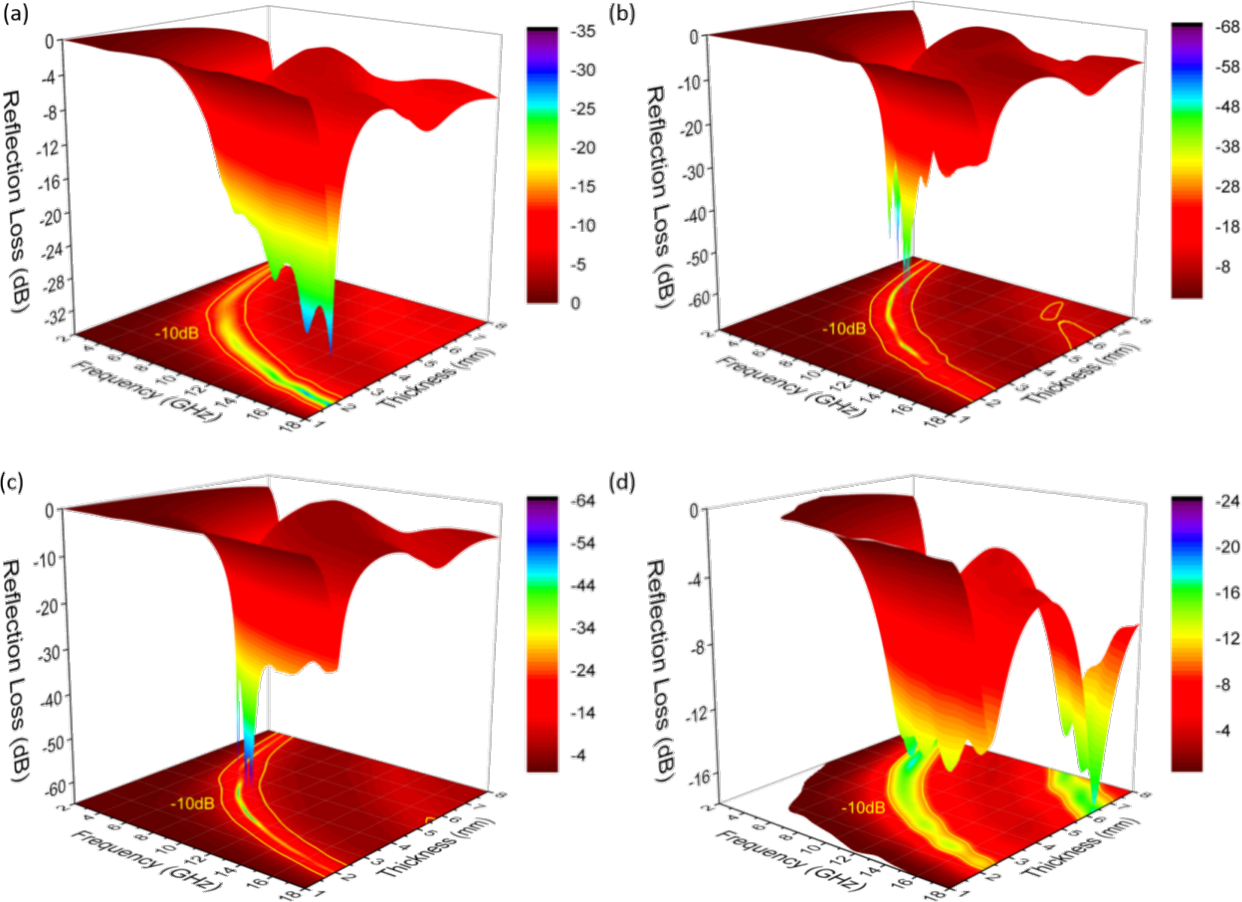
3D RL plots of the hybrids with different sample thicknesses in the range of 2–18 GHz with 30 wt % filler load. (a) SCZ0.5; (b) SCZ1; (c) SCZ2; (d) SCZ4.

The microwave absorption of the SCZ samples is simulated based on the transmission line theory (Figure 4). The reflection loss of the hybrid/wax mixtures as a function of the frequency is determined from *Z*_0_ and *Z*_in_ according to the following equation:


[1] $$\text{RL}\left(\text{dB}\right)=20\log\left|\frac{Z_{\text{in}}-Z_{0}}{Z_{\text{in}}+Z_{0}}\right|,$$

where *Z*_0_ and *Z*_in_ are the impedance of free space and the input impedance, respectively. Their relationship can be expresses as:


[2] $$Z_{\text{in}}=Z_{0}\sqrt{\frac{\mu_{\text{r}}}{\varepsilon_{\text{r}}}}\tanh\left(j\frac{2\pi fd}{c}\sqrt{\varepsilon_{\text{r}}\mu_{\text{r}}}\right),$$

where ε_r_ is the complex permittivity, ε_r_ = ε′ − *j*ε″, μ_r_ is the complex permeability, μ_r_ = μ′ − *j*μ″, *ƒ* is the frequency, *d* is the thickness of the material, and *c* is the speed of light.

The microwave absorption of the SCZ samples strongly depends on the dosage of ZnNO_3_·6H_2_O used for growing the ZnO particles. For SCZ0.5, the best RL_min_ value of −54.6 dB and an effective absorption bandwidth (EAB) of 6.6 GHz (11.36–17.96 GHz) are obtained for 25 wt % filler load at a sample thickness of 2.35 mm. For SCZ2 and SCZ4, the best RL_min_ values are, respectively, −63.9 dB and −58.1 dB, while their EAB values are 6.48 GHz (11.48–17.96 GHz) and 7.04 GHz (10.96–18.00 GHz), respectively. However, the filler loads should be increased to higher levels of 35–50 wt %. In general, SCZ1 exhibits the best performance among the samples. Its highest values of RL_min_ and EAB reach −65.4 dB and 7 GHz (10.96–17.96 GHz), respectively, at a small sample thickness and 30 wt % filler load. Furthermore, the EAB of other SCZ samples can even cover the X and Ku bands at small sample thicknesses (Supporting Information File 1, Table S1). These phenomena indicate that the EM absorption could be well controlled by adjusting the fractions of pristine materials and filler load of the absorbers. A comprehensive comparison with materials from our previous works (SiC@C and SiC@C-Fe_3_O_4_) and other reported materials (such as ZnO-decorated SiC_nw_ or graphene/SiC) [16,24,34–35] shows that the SCZ samples possess some advantages regarding lower RL_min_, broader EAB, and less filler load. This might enable a wide application as microwave absorbers as elaborated in this study.

**Figure 5:**
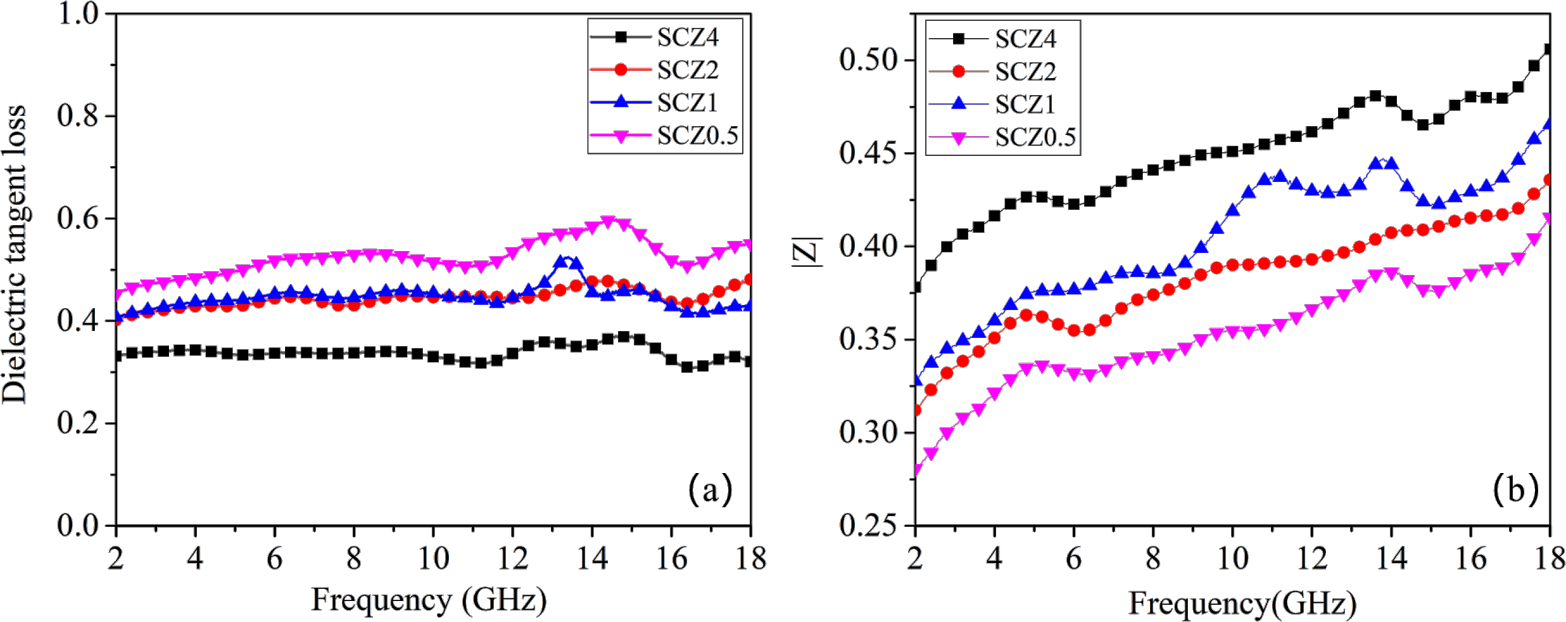
Dielectric tangent loss and impedance of SCZ/wax samples in the range of 2–18 GHz with a filler load of 30 wt %.

The dielectric behavior of the SCZ materials is analyzed to discover the reason for their outstanding performance. Figure 5 shows that the increase of the dosage of ZnNO_3_·6H_2_O does not lead to an increased dielectric tangent loss, suggesting that a moderate content of ZnO precursor is needed to synthesize SiC@C-ZnO with relatively good dielectric performance for microwave absorption. Although the SCZ0.5 sample has the highest value of dielectric tangent loss (Figure 5a), its impedance is not as high as that of the other SCZ samples (Figure 5b). A good EM absorber needs to exhibit high absorption and little reflection, which means an impedance matching is also necessary by controlling the values of ε′ and ε″. The permittivity values of the SCZ samples are shown in Figure S3 (Supporting Information File 1). The parameters ε′ and ε″ are all measured in the frequency range of 2–18 GHz using the coaxial wire method. The dielectric parameters of the samples gradually increase with increasing filler load, which is consistent with the effective medium theory [36]. Since the real part ε′ of the complex permittivity represents the capacity for storing electromagnetic waves and the imaginary part ε″ represents the loss of electromagnetic radiation, in general, ε′ and ε″ decrease with increasing dosage of ZnNO_3_·6H_2_O at the same filler load (20–50 wt %). This may be because both the real and imaginary parts of the carbon permittivity are higher than those of ZnO. Comparing the dielectric constant of SCZ0.5 and SiC@C at the same filler load of 30 wt %, the ε′ and ε″ values of SCZ0.5 are lower than the dielectric constants of SiC@C (Supporting Information File 1, Figure S4) [24].

Besides, the dielectric parameters greatly depend on the frequency (Supporting Information File 1, Figure S3). In general, a gradual decrease of ε′ and ε″ over almost the whole frequency range can be observed. It is to be noted that there are some fluctuations in the high-frequency range (10–16 GHz), which are called Debye relaxation peaks. These peaks are caused by shape anisotropy or surface polarization. For the SCZ samples, the unique core–shell structure and the interface polarization effect between different phases may account for this phenomenon. Actually, the dielectric relaxation process of electromagnetic waves in the SCZ samples can be well explained by the Debye theory [37]. According to this theory, the relationship between ε′ and ε″ can be expressed as:


[3] $${\left(\varepsilon^{\prime }-\frac{\varepsilon_{\text{s}}-\varepsilon_{\infty }}{2}\right)}^{2}+{\left(\varepsilon^{\prime \prime }\right)}^{2}={\left(\frac{\varepsilon_{\text{s}}-\varepsilon_{\infty }}{2}\right)}^{2},$$

where ε_s_ and ε_∞_ are the static and relative the dielectric permittivity at the high-frequency limit, respectively. Thus, the plot of ε′ and ε″ is a single semicircle, generally denoted as the Cole–Cole semicircle. At least one dielectric relaxation process occurs when a semicircle arises.

**Figure 6:**
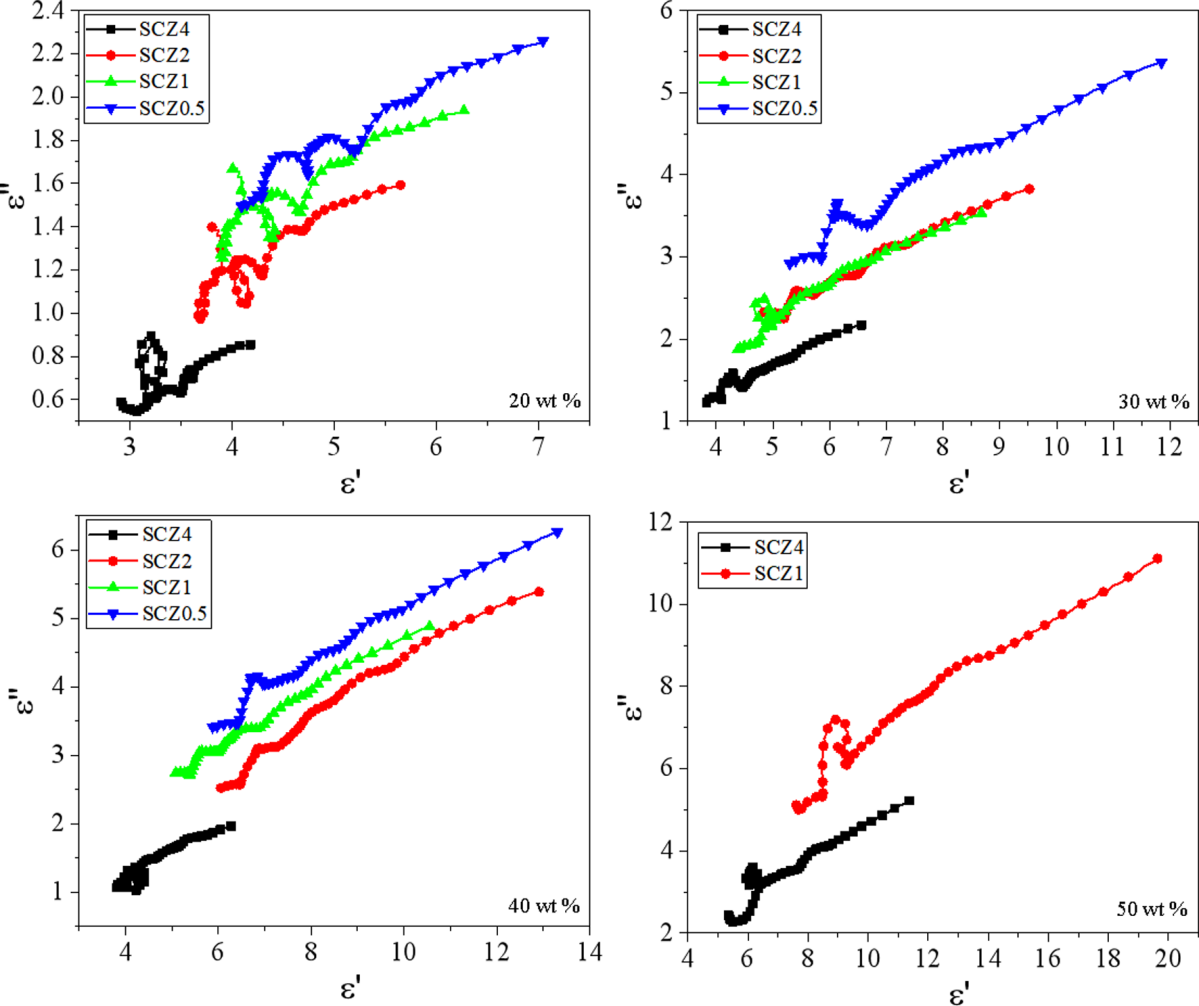
Plots of ε″ as a function of ε′ for all SCZ samples with different filler loads.

Figure 6 shows obvious semicircles under different conditions, especially at a filler load of 20 wt % for all SCZ samples. This indicates that multiple dielectric relaxation processes (such as Maxwell–Wagner relaxation and electron polarization) may occur when the electromagnetic waves interact with the materials [31]. The plots of ε′ and ε″ for all samples are quasi-linear, indicating that the conductivity loss through the carbon shell plays a dominant role in the EM dissipation.

**Figure 7:**
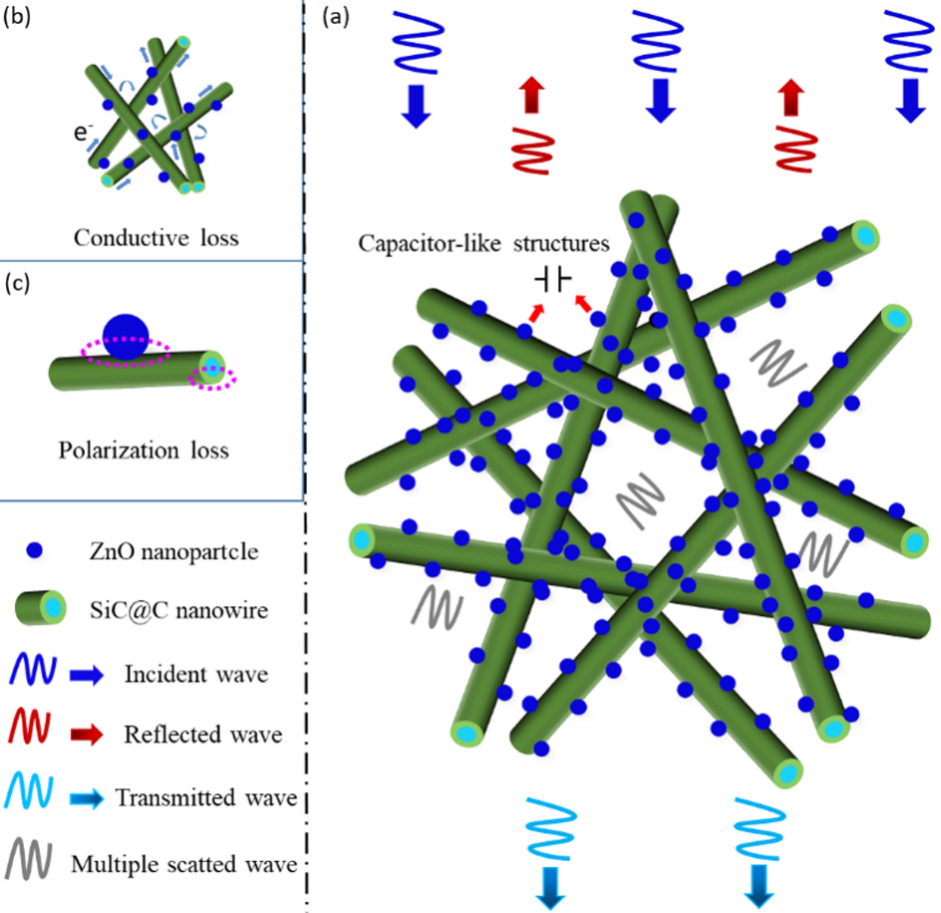
Microwave absorption mechanisms for the SCZ samples. (a) Capacitor-like structures in SiC@C-ZnO; (b) conductive loss in SiC@C-ZnO; (c) polarization loss in SiC@C-ZnO.

Based on the above analysis, it is considered that multiple loss mechanisms may contribute to the improvement of EM absorption for the as-prepared SiC@C-ZnO hybrids (Figure 7). First, the hybrid materials possess a large number of three-dimensional gaps, which are generated by the stacked one-dimensional SiC nanowires and the ZnO particles. These gaps can lead to reflection or scattering losses when the microwaves enter (Figure 7a). Second, conductivity losses can occur in the carbon shell on the SiC surface. As described in our previous work [24], the carbon shell may form a conductive network in the SCZ/wax composites (Supporting Information File 1, Figure S6b). Besides, abundant defects (such as nanopores in carbon) can also result in dipole polarization and Debye relaxations [24,38]. Furthermore, the introduction of immobilized ZnO particles on the carbon surface may result in the formation of capacitor-like structures at the heterogeneous interface between carbon and ZnO (Figure 7a) [31]. Also, the heterogeneous interface among the SiC_nw_ core, the porous carbon shell, and the ZnO particles possibly results in surface charge redistribution and generates interfacial polarization effects (Figure 7c). Liao et al. [39] reported a kind of multiphase nanocomposite (Co/ZnO/C) with porous structure for EM absorption. The best RL_min_ value of this material was −52.6 dB at a thickness of 3 mm at 12.1 GHz. They attributed the good performance to the synergy of several mechanisms, including multiple reflection and scattering losses generated by the porous structure and dielectric losses (such as interfacial polarization between ZnO and carbon or between Co and carbon, as well as between carbon and wax). These findings suggest that SiC@C-ZnO hybrids with diverse microstructures may have a bright future as EM absorbers.

## Conclusion

A new strategy for the controllable fabrication of SiC@C-ZnO hybrids via carbonization and hydrolysis reaction is described. Morphology and permittivity of the hybrids can be adjusted by changing the dosage of ZnNO_3_·6H_2_O. The amorphous carbon shell has a significant effect on the nucleation of crystalline ZnO particles, possibly due to oxygen-containing functional groups (such as carboxyl and hydroxy groups) and defects on the SiC@C surface, which both provide locations for the deposition of Zn^2+^ by electrostatic interactions. SCZ1 exhibits the best EM absorption properties. Its values for RL_min_ and EAB reach −65.4 dB and 7 GHz (10.96–17.96 GHz), respectively, at a small sample thickness and 30 wt % filler load. The effective EM absorption is related to the synergy of dielectric losses (including conductive loss and polarization relaxations) and multiple reflection or scattering losses, enabling a promising EM absorbing nanomaterial.

## Supporting Information

File 1 Additional experimental data.
