# Supplementary material for: ZnO-decorated SiC@C hybrids with strong electromagnetic absorption
Source: Beilstein J Nanotechnol. 2023 May 4;14:565–73. doi: 10.3762/bjnano.14.47 (PMC10167858; doi:10.3762/bjnano.14.47)
Supplement: File 1 — Additional experimental data. [file Beilstein_J_Nanotechnol-14-565-s001.pdf]

BEILSTEIN JOURNAL OF NANOTECHNOLOGY

Supporting Information

for

ZnO-decorated SiC@C hybrids with strong electromagnetic absorption

Liqun Duan, Zhiqian Yang, Yilu Xia, Xiaoqing Dai, Jian'an Wu and Minqian Sun

Beilstein J. Nanotechnol. **2023,** *14,* 565–573. doi:10.3762/bjnano.14.47

Additional experimental data

License and Terms: This is a supporting information file under the terms of the Creative Commons Attribution License (https://creativecommons.org/licenses/by/4.0). Please note that the reuse, redistribution and reproduction in particular requires that the author(s) and source are credited and that individual graphics may be subject to special legal provisions.
The license is subject to the *Beilstein Journal of Nanotechnology* terms and conditions: (https://www.beilstein-journals.org/bjnano/terms)

Experimental

Pristine materials

Pristine SiC_{nw} (diameter: 100–600 nm; length >100 μm; density: 3.21 g/cm^3; purity: ≈98%) was purchased from XF Nano Materials Tech Co., Ltd. (Nanjing, China).

Characterization and measurements

The morphology of the SCZ materials was characterized through transmission electron microscopy (TEM) on a FE-HRTEM, Tecnai G2 F20UTwin (FEI, USA) at 200 kV. X-ray photoelectron spectroscopy (XPS) experiments were carried out on a K-Alpha 1063 system (Thermo Fisher Scientific). The X-ray anode was run at 72 W and the high voltage was kept at 12.0 kV. The base pressure of the analyzer chamber was about 1×10^{-9} mbar. A whole spectrum scan (0–1400 eV) and regional scans of all the elements at very high resolution were recorded for each sample. X-ray diffractometry (XRD) was carried out on a Bruker D8 advance (Bruker, Germany). Relative complex permittivity (ε_r) and permeability (μ_r) in the 2–18 GHz range were obtained through a vector network analyzer (N5242A PNA-X, Agilent, UK). Uniform mixtures of SCZ samples and wax were pressed into a toroidal shape (outer diameter: 7.00 mm, inner diameter 3.04 mm).

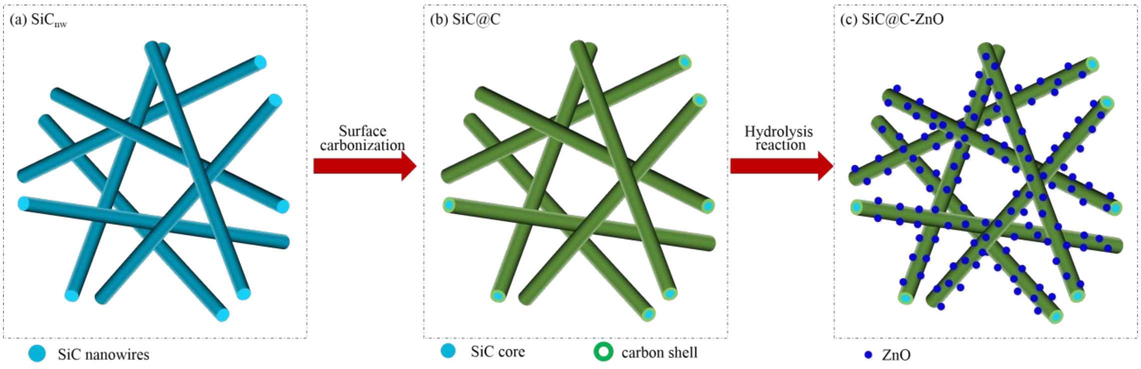

Figure S1: Schematic illustration of the fabrication of SCZ hybrids.

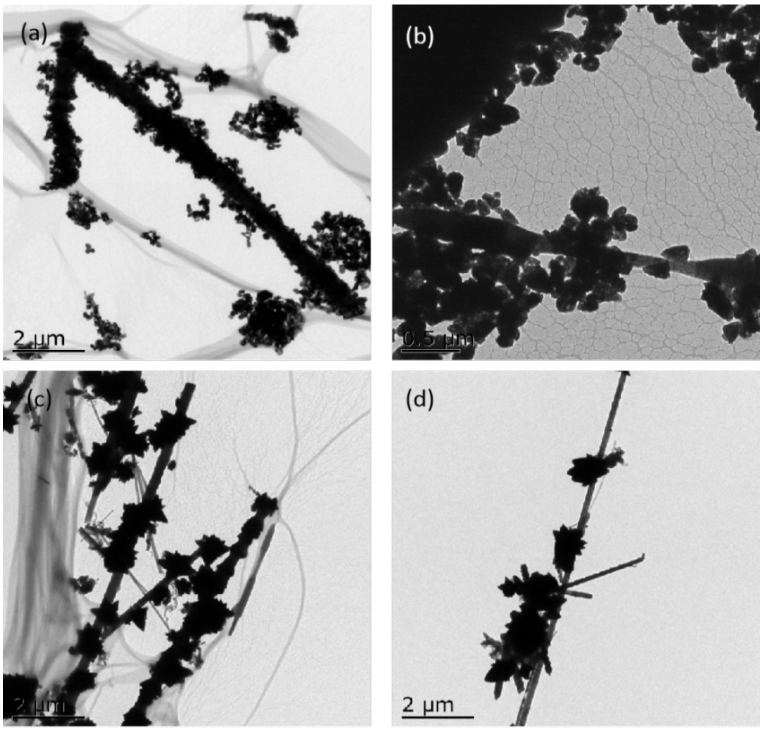

Figure S2: TEM images of SCZ samples. (a) SCZ4; (b) SCZ3; (c) SCZ2; (d) SCZ1.

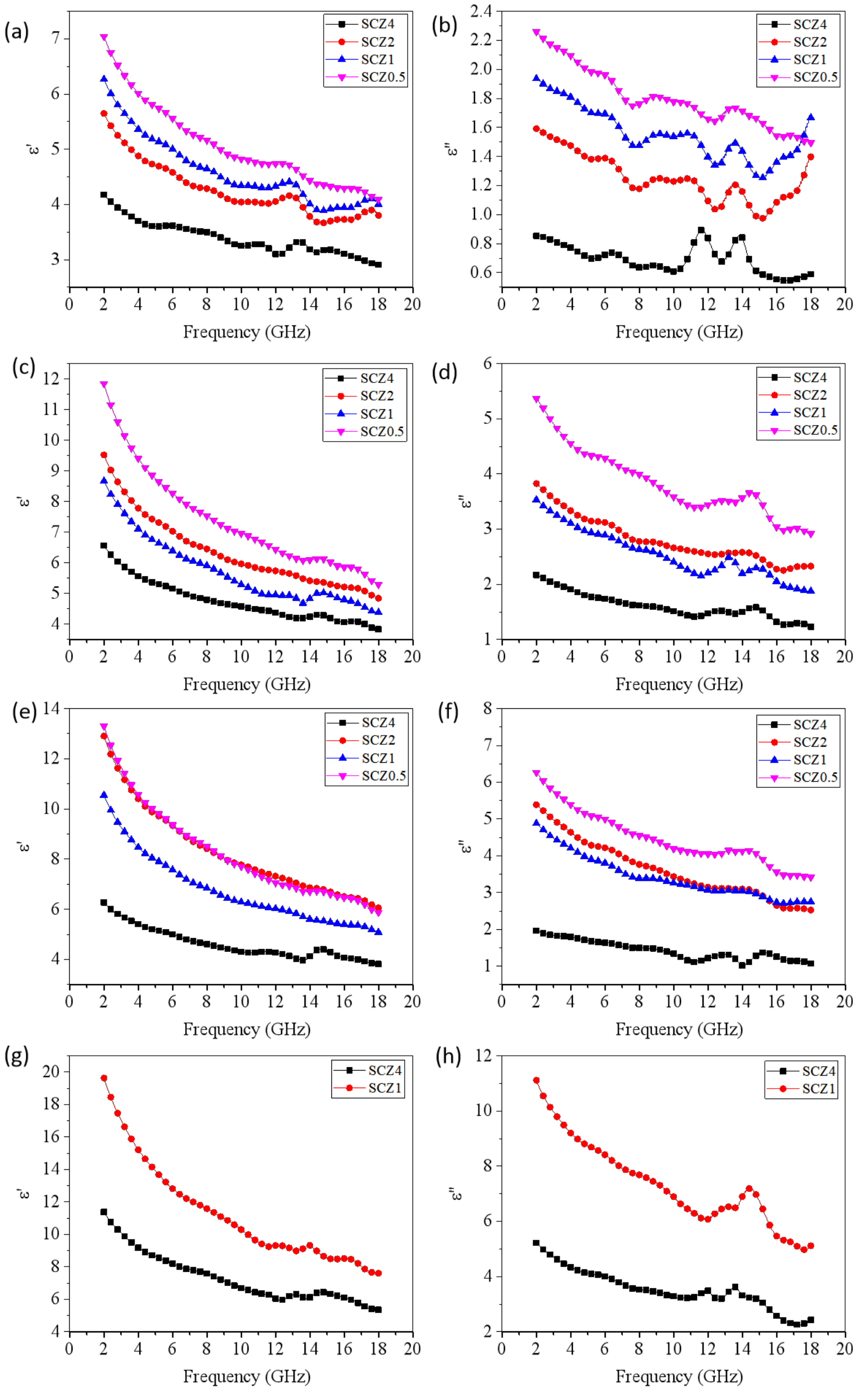

Figure S3: Real part (a,c,e,g) and imaginary part (b,d,f,h) of the relative complex permittivity of SCZ samples with different loadings. (a, b) 20 wt %; (c, d) 30 wt %; (e, f) 40 wt %; (g, h) 50 wt %.

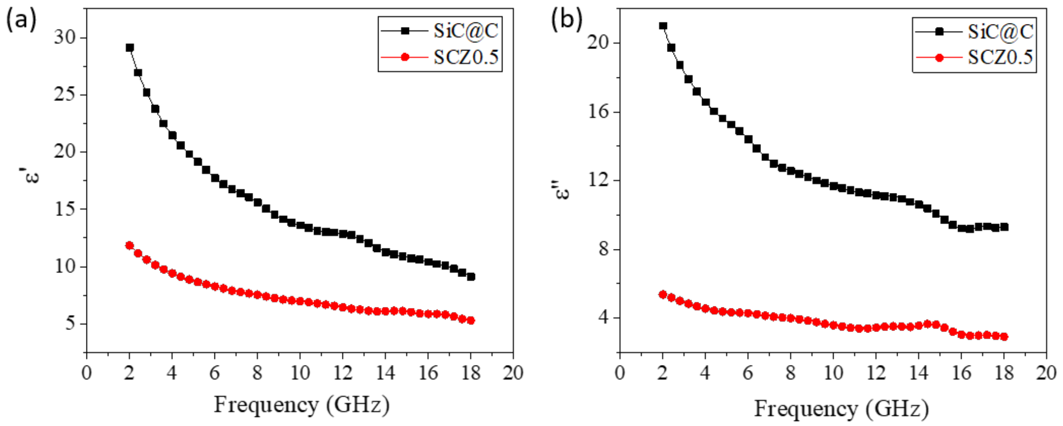

Figure S4: Real part (a) and imaginary part (b) of the relative complex permittivity of SCZ0.5 and SiC@C samples.

Table S1: The minimum matching thickness of SCZ samples for covering X and Ku bands.

Samples	Filler load (wt %)	d_x (mm)[a]	d_{Ku} (mm)[b]
SCZ0.5	30	2.98	2.15
SCZ0.5	40	2.85	2.09
SCZ1	30	3.47	2.44
SCZ1	40	3.16	2.88
SCZ2	30	3.28	2.35
SCZ2	35	—	2.2
SCZ2	40	2.82	—
SCZ4	50	3.03	2.08

[a]d_x and d_{Ku} indicates the minimum matching thickness for the X and Ku bands, respectively.
